# Evaluation of the MoMba Live Long Remote Smoking Detection System During and After Pregnancy: Development and Usability Study

**DOI:** 10.2196/18809

**Published:** 2020-11-24

**Authors:** Stephanie Valencia, Laura Callinan, Frederick Shic, Megan Smith

**Affiliations:** 1 Child Study Center Yale University School of Medicine New Haven, CT United States; 2 Carnegie Mellon University Pittsburgh, PA United States; 3 Department of Psychiatry Yale University School of Medicine New Haven, CT United States; 4 Seattle’s Children’s Research Institute University of Washington School of Medicine Seattle, WA United States

**Keywords:** breath carbon monoxide, contingency management, smoking cessation, pregnancy, mobile-based sensor, mobile phone

## Abstract

**Background:**

The smoking relapse rate during the first 12 months after pregnancy is around 80% in the United States. Delivering remote smoking cessation interventions to women in the postpartum period can reduce the burden associated with frequent office visits and can enable remote communication and support. Developing reliable, remote, smoking measuring instruments is a crucial step in achieving this vision.

**Objective:**

The study presents the evaluation of the MoMba Live Long system, a smartphone-based breath carbon monoxide (CO) meter and a custom iOS smartphone app. We report on how our smoking detection system worked in a controlled office environment and in an out-of-office environment to examine its potential to deliver a remote contingency management intervention.

**Methods:**

In-office breath tests were completed using both the MoMba Live Long system and a commercial monitor, the piCO^+^ Smokerlyzer. In addition, each participant provided a urine test for smoking status validation through cotinine. We used in-office test data to verify the validity of the MoMba Live Long smoking detection system. We also collected out-of-office tests to assess how the system worked remotely and enabled user verification. Pregnant adult women in their second or third trimester participated in the study for a period of 12 weeks. This study was carried out in the United States.

**Results:**

Analyses of in-office tests included 143 breath tests contributed from 10 participants. CO readings between the MoMba Live Long system and the piCO^+^ were highly correlated (*r*=.94). In addition, the MoMba Live Long system accurately distinguished smokers from nonsmokers with a sensitivity of 0.91 and a specificity of 0.94 when the piCO^+^ was used as a gold standard, and a sensitivity of 0.81 and specificity of 1.0 when cotinine in urine was used to confirm smoking status. All participants indicated that the system was easy to use.

**Conclusions:**

Relatively inexpensive portable and internet-connected CO monitors can enable remote smoking status detection in a wide variety of nonclinical settings with reliable and valid measures comparable to a commercially available CO monitor.

**Trial Registration:**

ClinicalTrials.gov NCT02237898; https://clinicaltrials.gov/ct2/show/NCT02237898

## Introduction

### Background

Tobacco is the most common substance of abuse used during pregnancy, with a substantially higher rate of use among socioeconomically disadvantaged women [1]. While approximately 50% of women who previously smoked regularly abstain from smoking in pregnancy, relapse to smoking postnatally remains a challenge, with over 70% of women who remitted relapsing within 12 months postpartum in the United States [2,3].

Contingency management (CM), rewarding financial incentives contingent upon biochemically verified abstinence from recent smoking, has consistently been shown to decrease the use of tobacco [2-4] and to be an efficacious intervention for promoting smoking cessation in pregnant and postpartum women [5]. Delivering CM interventions typically involves frequent monitoring of smoking status with daily or weekly office visits. In this work, we develop a remote smoking assessment system to more widely disseminate smoking cessation interventions into community settings and to reach overburdened and underserved populations of smokers, such as expectant mothers of lower socioeconomic status.

### Technologies for Remote Smoking Assessments

Mobile technologies, especially web-enabled or smartphone technology, can be used to access real-world community settings, as the access to smartphones has increased: 81% of Americans owned a smartphone in 2019 [6]. Mobile health solutions are emerging, creating an opportunity for the expansion of evidence-based practices [7-9]. In combination with sensors that enable remote biochemical assessment of smoking status, such as breath carbon monoxide (CO) measurements [10-12], mobile technologies could allow remote delivery of smoking cessation interventions.

A randomized, controlled, parallel-group design study carried out a 6-week intervention evaluating the effectiveness of an internet-based smoking cessation program in the United States using a system called Motiv8 [13]. Participants used Motiv8 to submit videos and values of breath CO tests taken with a commercial CO breath analyzer, the piCO^+^ Smokerlyzer (Bedfont Scientific), and to confirm identity and veracity of tests through a secured website. Even with some limitations (eg, the need for a desktop computer), the study demonstrated that participants receiving rewards based on abstinence were more likely to post negative CO samples on the website than the participants who received monetary rewards independent of smoking status (odds ratio 4.56, 95% CI 2.10-9.52). More recently, breath CO manufacturers have developed commercial CO monitors that leverage the advantages of internet-based systems with new, smaller CO monitors such as the iCO Smokerlyzer (Bedfont Scientific). The iCO Smokerlyzer connects to a smartphone for personal smoking behavior monitoring and enables users to measure their CO levels but does not include a way to verify that the intended user completed the breath test appropriately. This verification is important for remote CM interventions, where additional evidence is needed to verify test validity as there are no present observers. Recent work has investigated unsupervised breath test validity by verifying exhalation through the use of pressure sensors and the use of facial recognition for user authentication in the context of smoking and alcohol use [14-16].

### The MoMba Live Long System

#### Overview

**Figure 1:**
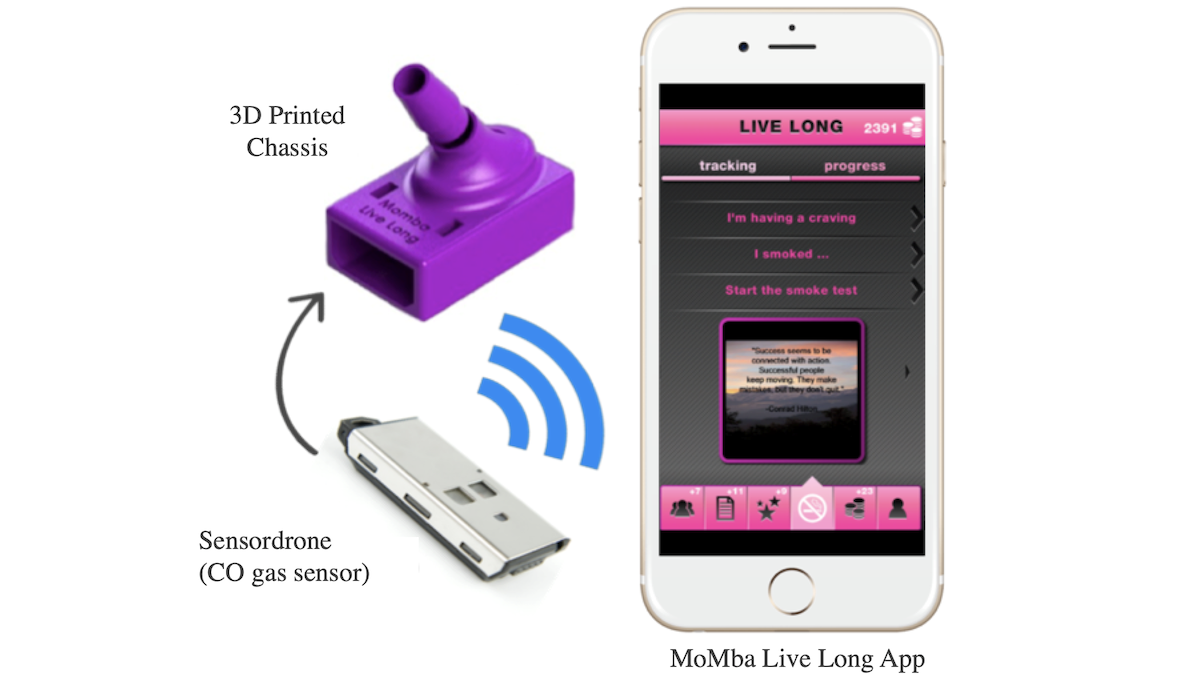
The custom and portable breath carbon monoxide (CO) meter is comprised of an environmental CO gas sensor and a custom 3D-printed chassis. A smartphone was loaded with a custom app (MoMba Live Long) that enables participants to complete scheduled breath tests and collect rewards. The Sensordrone and chassis sizes are shown at scale with respect to the phone in the image.

The MoMba Live Long system consisted of a custom and portable breath CO meter that wirelessly interfaced via Bluetooth with an iOS-only app (see Figure 1). The MoMba Live Long iOS app provided an interface for the breath CO meter that allowed the participant to receive notifications regarding the availability of the breath test, taking the breath test, seeing results of smoking status, and keeping track of rewards and progress. In addition, the app verified that the participant was correctly taking the breath test by recording pictures using the front-facing camera as well as recording audio to verify that the participant was exhaling while taking the test. When a breath test indicated smoking abstinence, the participant was rewarded with tokens that could be exchanged for gift cards. The MoMba Live Long app also enabled the delivery of questionnaires through the app. The MoMba Live Long system was based on a successful app design developed to support maternal mental health in the postpartum period [17].

#### MoMba Breath CO Meter

The custom breath CO meter used an electrochemical CO gas sensor—the Sensordrone, part No. SDRONEG1 (Sensorcon Inc)—designed to measure environmental CO. The Sensordrone was accurate to within 10% with a resolution of 1 parts per million (ppm), making it comparable to existing commercial medical-grade CO breath analyzers [18]. We designed a 3D-printed chassis to encase the Sensordrone and allow proper air flow. 3D printing was selected as a fabrication method that allowed us to prototype in a timely manner and test different designs. The 3D-printed chassis held an inline activated-carbon filter and cotton that removed non-CO gases and excess moisture.

#### Breath Sample Collection

To determine smoking status, a user completed a breath test by blowing into our custom breath CO meter composed of a disposable mouthpiece connected to the custom 3D-printed chassis encasing the Sensordrone. The breath CO meter measured CO concentration in the breath after collecting exhaled air samples for 20 seconds at a 5-Hz sampling rate. Collected data were securely sent and stored in a back-end server monitored by staff.

#### The MoMba CO Estimate

A previous study we conducted established that the most accurate prediction of smoking status from breath was by sampling the portion of breath at the end of exhalation, which represented the middle portion of an exhalation of 20 seconds, on average [19]. During this initial testing, we detected baseline reading offsets attributed to increased temperature in the Sensordrone from recent charging and environmental pollution. This led to the addition of a baseline correction to our algorithm. Our previous findings presented an exhaustive exploration of different smoking detection approaches using different classification models and features, while this report presents an evaluation of our system’s performance with our implemented algorithm that used the best-performing features from our previous study [19]. In order to obtain one single value from a 20-second sample of breath tests, we performed the following steps: (1) calculated the baseline reading for the Sensordrone, (2) located the general maximum and captured values above 50% of the maximum value, and (3) calculated the median value and removed any offsets detected in the baseline value. This final value represented the MoMba CO estimate.

### Aim of the Study

We present the design of the MoMba Live Long , mobile, breath CO meter and a pilot evaluation of the feasibility of the system as a smoking assessment tool during and after pregnancy.

## Methods

### Participants

The MoMba Live Long pilot study was approved by the ethical review board of the Yale School of Medicine. Participants were recruited at local clinics; through community outreach, flyers, and advertisements; and from referrals. Participants were women who met the following inclusion criteria: were 18 years of age or older, were daily tobacco cigarette smokers not using nicotine replacement therapy, were pregnant with a singleton in their second or third trimester, and had a desire to stop smoking. Women were not eligible if English was not their primary language, if they did not live in the city in which the study was conducted, if they did not plan to deliver their baby at the local hospital, or if they met medical exclusions, as determined by medical record review, including respiratory medical conditions such as chronic obstructive pulmonary disease, HELLP (hemolysis, elevated liver enzymes, low platelet count) syndrome, or pregnancy complications. If a participant lost her child, she was no longer eligible to participate. Eligible participants completed an intake visit where informed written consent was provided and smoking status was confirmed with a urine sample.

### In-Office and Remote Data Collection Procedures

During the MoMba Live Long pilot study, breath tests were collected for 12 weeks and followed the CM schedule described by Higgins et al [20]. For each consecutive smoke-free breath test, a participant received a higher number of tokens per test. Breath samples were obtained via a mixture of in-office and remote tests. Each participant was asked to attend up to 18 in-office visits: five visits in week 1, two visits in weeks 2 and 3, and one visit per week for weeks 4-12. In-office visits did not always coincide with a CM-related breath test. Before completing breath tests, participants were asked to report the number of cigarettes smoked within the previous 24 hours and the date and time of the last cigarette smoked through the MoMba Live Long app. In-office visits consisted of first completing one breath test using the MoMba Live Long system and then one breath test with piCO^+^. Participants also provided a urine sample to validate smoking.

During in-office testing, participants were asked to sit in a chair in an upright and straight position to accurately complete a breath test. Participants were instructed to inhale deeply and hold their breath for 15 seconds and then exhale all the air completely (up to 20 seconds). Holding the breath allowed the sensor to obtain a CO value close to alveolar CO concentration [21]. Both the MoMba Live Long system and the piCO^+^ monitor displayed a countdown on their respective screens as participants were holding their breath. Once the countdown was finished, the participant blew into the sensor. Both systems displayed a second countdown to indicate that the sensor was collecting the sample.

All remote tests followed the same procedures as in-office breath tests. The participants received a kit to take home that included an iPhone, which participants were encouraged to use as their primary phone; a chassis; a Sensordrone; a charger for the Sensordrone; disposable mouthpieces; and replacement filters. After participants completed their tests, research staff verified the validity of the test using the back-end server and approved the corresponding financial rewards. Participants received a notification to complete their test between the hours of 8:30 AM and 3 PM EST. The notification for the breath test expired after 5 hours.

### Measures

#### Participant Characteristics at Intake

A baseline questionnaire asked questions regarding demographics and smoking habits. The Fagerstrom Test for Cigarette Dependence was used to assess dependence on nicotine; a higher score indicates greater dependence [22].

#### MoMba Performance Against Gold-Standard Measures

The primary outcome measure for this study was the validity of the MoMba CO outcome; this measure was compared with the gold standard of piCO^+^ and urine cotinine collected at the same visit. A measure of smoking abstinence was defined as CO-negative breath samples determined by the MoMba breath CO meter and the piCO^+^ (≤6 ppm). The selected CO cutoff levels were recommended by previous work [10,23]. All urine tests were tested for adulteration using a specimen validity test and then sent to a lab for a quantitative cotinine urine assay with quality control. A cotinine concentration value less than 50 ng/mL indicated smoking abstinence [24]. Due to limits in lab detection, cotinine concentrations reported as less than 10 ng/mL were estimated as 5 ng/mL.

#### Variation of CO Values Since Last Cigarette Smoked

To examine how breath CO values varied since last cigarette smoked, the half-life of breath CO, 3-6 hours [12], was used to create a dichotomous variable indicating if the participant smoked within 5 hours of a breath test or more than 5 hours before a breath test.

#### Variation of CO Values According to Pregnancy Status

Pregnancy and postpregnancy CO values were compared to explore any potential differences; postpregnancy status was determined by a participant’s delivery date. We used the number of cigarettes and time since last cigarette smoked to investigate if these variables impacted the observed pattern of results.

#### Out-of-Office Performance and User Experience

A follow-up questionnaire was asked 3 months after intake, at the completion of CM; this questionnaire included two Likert scale questions regarding how easy it was to use (1) the sensor and (2) the MoMba Live Long app; response options were 1 (Extremely Difficult), 2 (Difficult), 3 (Neutral), 4 (Easy), and 5 (Extremely Easy). In addition, we evaluated the completion and delivery of the remote breath test as well as the performance of the front-facing camera authentication method and the microphone to detect exhalation during remote breath tests.

### Statistical Analysis

Categorical variables were described with count and proportions. Normally distributed data were presented with mean and SD; nonnormally distributed data were presented with median and IQR. Sensitivity was defined as the proportion of tests for which a true-positive breath CO test was detected. Specificity was defined as the proportion of tests for which there was a true-negative breath CO test. Receiver operating characteristic curves were generated for the MoMba CO estimate and the piCO^+^ by plotting the percentage of true positives against the percentage of false positives. Area under the curve (AUC) was calculated for each plot using the pROC package for R (The R Foundation) [25]. Since each participant contributed multiple observations, methods to calculate sensitivity and specificity accounted for clustering due to participant [26]. To assess the relationship across smoking measures, a correlation coefficient was calculated accounting for repeated observations of participants [27]. Generalized linear models were used to compare smoking status indicators between groups, accounting for potentially correlated data among participants. An autoregressive correlation structure was specified for the models. Time since last cigarette smoked and/or number of cigarettes smoked in the past 24 hours were used as covariates in the model. Statistical significance was determined as *P*<.05 (2-tailed).

## Results

### Participant Characteristics at Intake

A total of 10 pregnant adult women participated in the MoMba Live Long pilot. The majority of women were single and never married (7/10, 70%); 50% (5/10) of the women were Black or African American, non-Hispanic (see Table 1). The average age of participants was 31.7 years (SD 4.6).

**Table 1:** Demographics and clinical characteristics of participants from the MoMba Live Long pilot study.

Characteristic		Value (N=10)
**Marital status,** **n (%)**		
	Single and never married	7 (70)
	Married or partnered	3 (30)
**Race and ethnicity,** **n (%)**		
	White, non-Hispanic	4 (40)
	Black or African American, non-Hispanic	5 (50)
	White, Hispanic	1 (10)
**Highest year of education completed,** **n (%)**		
	Grades 9-12 or General Educational Diploma	5 (50)
	At least 1 year of college or vocational school	5 (50)
**How many cigarettes per day do you smoke? n (%)**		
	0-5 cigarettes	0 (0)
	6-10 cigarettes	9 (90)
	11-20 cigarettes	1 (10)
Age in years, mean (SD)		31.7 (4.6)
Previous pregnancies not including current pregnancy, mean (SD)		3.7 (2.5)
Number of weeks pregnant, mean (SD)		25.9 (9.1)
Fagerstrom score^a^, mean (SD)		4.5 (1.6)

^a^Fagerstrom Test of Cigarette Dependence conducted at intake; scores range from 1 to 10.

### MoMba Performance Against Gold-Standard Measures

A total of 143 breath tests were collected; participants contributed, on average, 14.3 (SD 3.5) tests (range 8-17). Significant CO reading correlations (*r*=.94) were observed between the MoMba CO estimate and the piCO^+^ (see Table 2). When using the piCO^+^ as the gold standard, the MoMba CO estimate presented a sensitivity of 0.91 and specificity of 0.94.

Of the 139 tests with urine data, a moderate linear relationship was seen between both CO breath measures and the urine cotinine tests (see Table 2): MoMba CO estimate (*r*=.52) and piCO^+^ (*r*=.57). When using cotinine in urine as the gold-standard smoking indicator, the sensitivity with the MoMba CO estimate was 0.81 and the piCO^+^ measure showed a sensitivity of 0.87. Both MoMba CO and piCO^+^ estimates had a specificity of 1.0.

**Figure 2:**
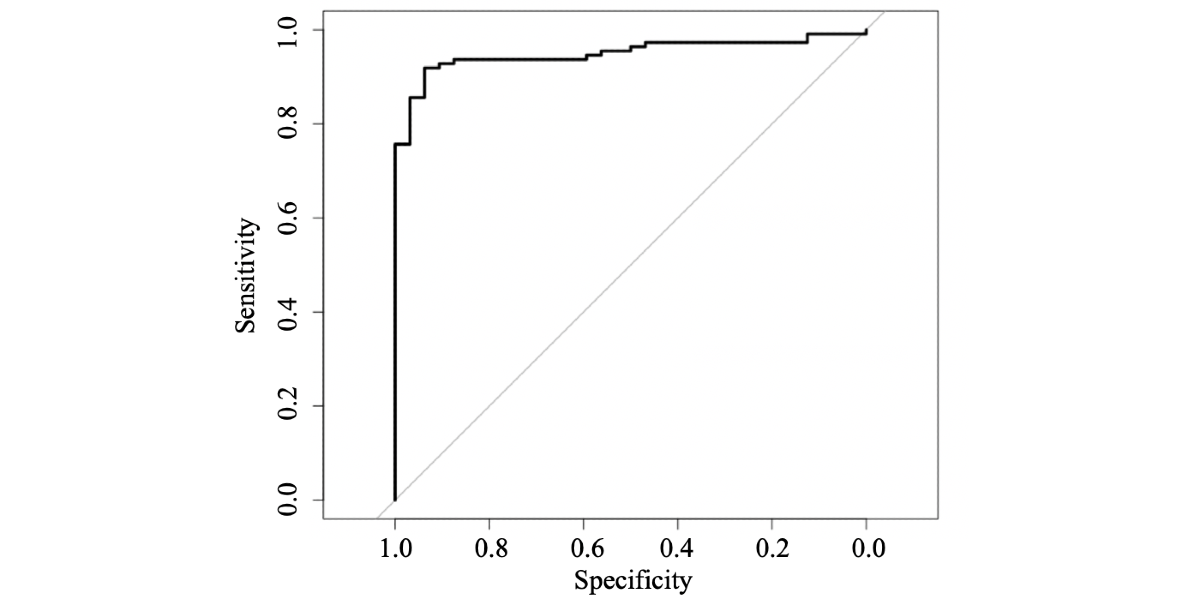
Receiver operating characteristic curves for the MoMba Live Long system carbon monoxide (CO) measure with the piCO^+^ measure as gold standard.

**Figure 3:**
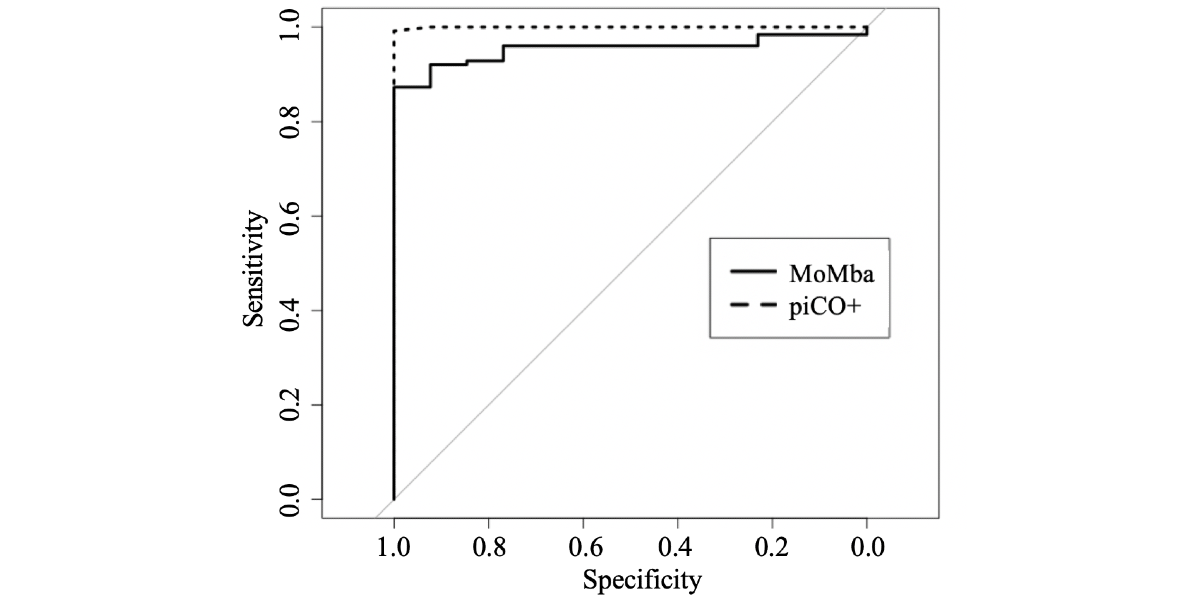
Receiver operating characteristic curves for the MoMba Live Long system carbon monoxide (CO) and piCO^+^ measures with cotinine in urine as gold standard.

The AUC for the MoMba CO estimate was 0.95 (95% CI 0.91-0.99) when the piCO^+^ measure was used as a gold standard (see Figure 2). With urine cotinine as the gold standard, the AUC for the MoMba CO estimate was 0.95 (95% CI 0.92-0.99) and for the piCO^+^ measure was 0.99 (95% CI 0.99-1.0) (see Figure 3).

**Table 2:** Sensitivity and specificity of the MoMba Live Long system carbon monoxide (CO) measure with the piCO^+^ measure as gold standard, and MoMba Live Long system CO and piCO^+^ measures with cotinine in urine as gold standard.

Smoking status test		Sensitivity (95% CI)	Specificity (95% CI)	*r*	*P* value
**piCO^+^ as gold standard**					
	MoMba CO estimate (n=143)	0.91 (0.80-1.0)	0.94 (0.83-1.0)	.94	<.001
**Cotinine in urine as gold standard**					
	MoMba CO estimate (n=139)	0.81 (0.65-0.97)	1.0 (1.0-1.0)	.52	.01
	piCO^+^ (n=139)	0.87 (0.73-1.0)	1.0 (1.0-1.0)	.57	.02

### Variation of CO Values Since Last Cigarette Smoked

**Figure 4:**
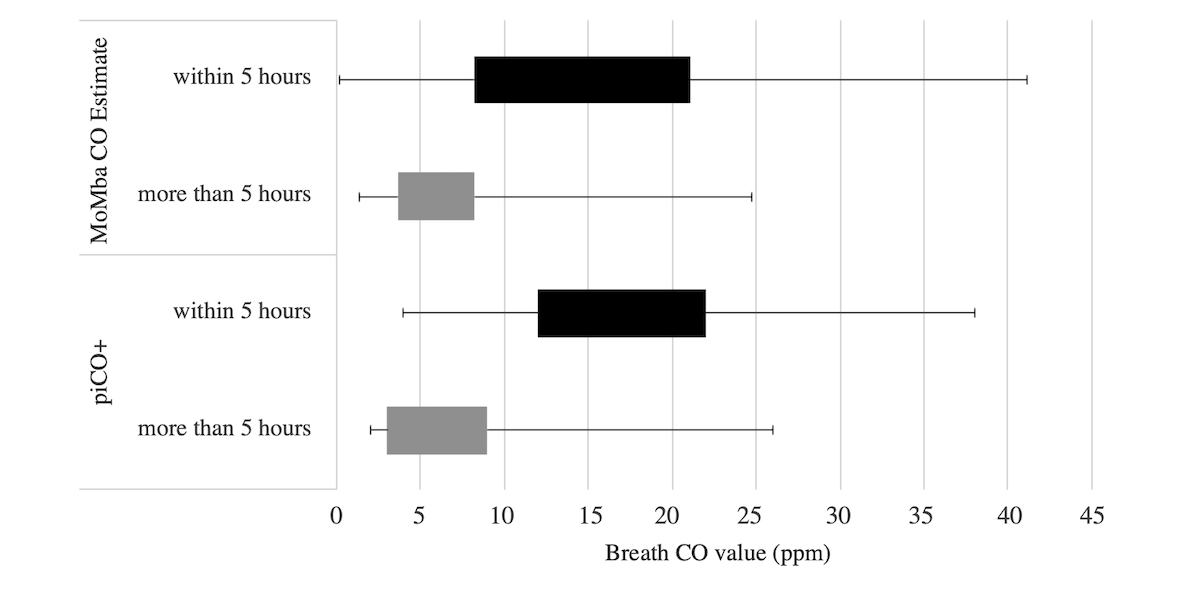
Breath carbon monoxide (CO) values according to time since last cigarette smoked. ppm: parts per million.

Figure 4 shows the difference in CO measures based on the dichotomized variable *time of last cigarette smoked*. Of the 143 completed in-office breath tests, 8 (5.6%) tests did not have data regarding time of last cigarette smoked, 102 (71.3%) breath tests were taken within 5 hours of smoking, and 33 (23.1%) tests were taken after 5 hours of smoking. All breath CO values were higher when a participant smoked within 5 hours of the breath test (MoMba CO estimate, *P*=.048; piCO^+^, *P*=.03). After controlling for the number of cigarettes smoked in the past 24 hours, values remained higher for participants who smoked within 5 hours of the test compared with participants who smoked more than 5 hours before the test (MoMba CO estimate, *P*=.045; piCO^+^, *P*=.02).

### Variation of CO Values According to Pregnancy Status

**Figure 5:**
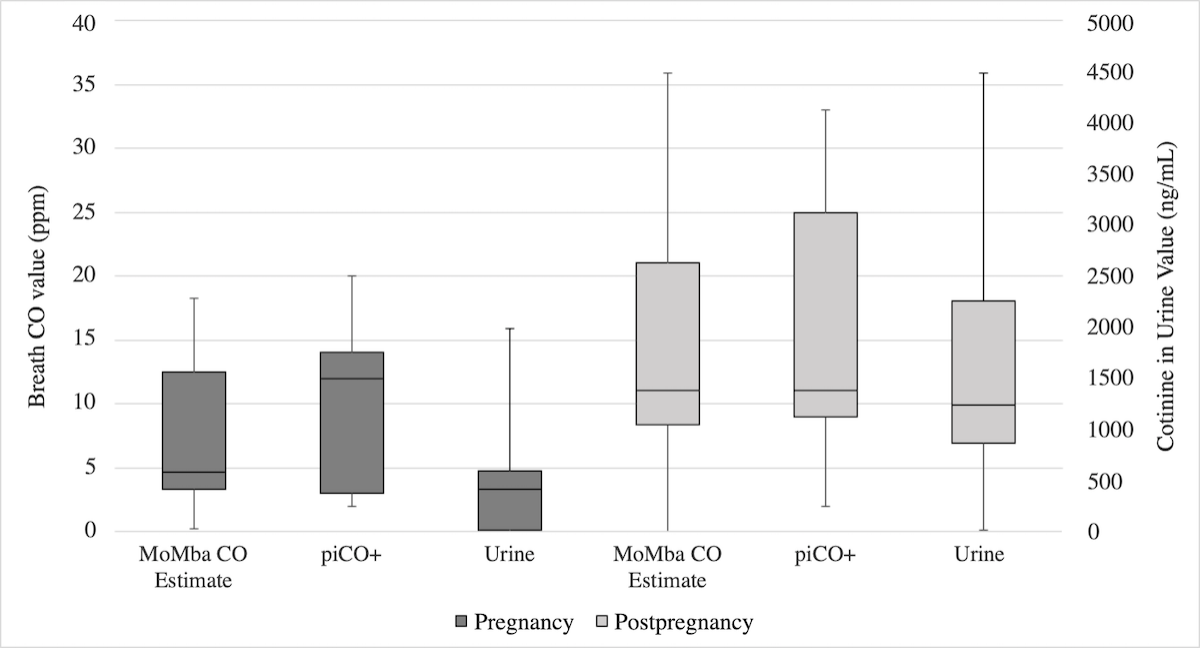
Breath carbon monoxide (CO) and cotinine in urine values according to pregnancy status (n=4). ppm: parts per million.

Among the 4 participants who delivered a baby during the study (65 breath tests), the median MoMba CO estimate was 4.7 ppm (IQR 3.3-12.5) during pregnancy and 11.1 ppm (IQR 8.4-21.0) after pregnancy (see Figure 5). The median CO estimate from piCO^+^ was 12 ppm (IQR 3-14) during pregnancy and 11 ppm (IQR 9-25) after pregnancy. There were no differences between values during pregnancy compared with values after pregnancy for all three smoking indicators; this remained after controlling for number of cigarettes smoked in the past 24 hours and time of last cigarette smoked.

### Out-of-Office Performance and User Experience

Of the 102 remote breath tests successfully sent to participants, 51 (50.0%) tests were completed. Participants received, on average, 10 remote breath test notifications (SD 3.6) and completed 5 breath tests on average (SD 3.5). Almost all remote breath tests (47/51, 92%) indicated smoking. Remote breath tests that were not completed were preceded by a breath test indicating smoking 88% (45/51) of the time; average time from a missed remote test to the preceding completed breath test was 9.8 days (SD 7.2). Notifications were not successfully delivered for 7 out of 51 (14%) remote tests; in 2 of these instances (29%), the participant received a replacement breath test in the office, and in 2 instances (29%) a replacement breath test was sent remotely to the participant. Out of 51 remote breath tests, 4 (8%) results were challenged by a participant; research staff examined the data to determine if there was an inconsistency in breath sample values and pictures. If results were indicative of a false positive, the participant was sent a new breath test.

All 51 completed remote breath tests had pictures where staff could verify the identity of the participant completing the test; only one set of pictures for 1 remote breath test out of 51 (2%) did not show the participant’s mouth. All participants used the front-facing camera feature correctly. Audio was available for all remote breath tests except for 1 out of 51 (2%), in which the file was corrupted. About 50% of audio recordings also included background noise, making it difficult to detect the sounds of breath; these background sounds included TV, radio, fans, babies, and other people talking.

At the 3-month follow-up, 63% (5/8) of participants indicated that the MoMba Live Long app and the Sensordrone were “extremely easy to use,” and 38% (3/8) indicated that it was “easy to use.”

## Discussion

### Principal Findings

In this study, we tested and validated the MoMba Live Long system’s breath CO meter as a smartphone-based system that can determine smoking status during and after pregnancy. Collected breath CO values with the MoMba Live Long system compared well to the commercial piCO^+^ monitor. Women in our study found the system easy to use. Regarding the novelty of our system, in comparison with other remote smoking measurement instruments, including the piCO^+^ and the new iCO^+^ Smokerlyzer (Bedfont Scientific), the MoMba Live Long CO meter has several advantages: (1) it interfaces wirelessly with a smartphone app, (2) it enables user verification, through picture and audio capturing for smoke test validity on the go, and (3) it can detect smoking status during and after pregnancy. Further investigation is required to determine what additional system features are needed to make home CM interventions feasible beyond ensuring the accuracy of the measurement system.

### System Performance Metrics in the CM Context

When delivering financial incentives through remote verification of smoking status, specificity—the proportion of tests for which there is a true-negative breath CO test—is the measurement that should be prioritized to positively reinforce participants who have abstained from smoking. The MoMba Live Long system achieved high levels of specificity; false positives in the context of a CM intervention can decrease motivation to quit and increase frustration for participants.

### Breath CO as a Time-Dependent Marker for Smoking

While CO concentration and cotinine can both measure the presence or absence of smoking, the difference in the half-lives of these analytes do not make them directly comparable. CO has a shorter half-life (3-6 hours) in comparison to cotinine (17 hours in nonpregnant women and 9 hours in pregnant women [28]). This difference helps explain the finding of an observed, moderate, linear relationship between both the MoMba CO estimate and the piCO^+^ measure with urine cotinine tests. CO concentration in the breath can be a better measure of cigarette consumption within a shorter period of time than cotinine, as shown in previous work [29], making it suitable for recent and immediate smoking assessments [12]. Furthermore, our analysis found differences in breath CO levels within the 5 hours of the last cigarette smoked and after the 5-hour mark, suggesting that future remote CM interventions should consider sampling more than one time a day.

### The Need for Context-Aware CO Cutoffs

Prior work has suggested a variety of optimal cutoff breath CO levels to determine smoking status [12,24,30-32]. While we did not observe differences in CO values during pregnancy and the postpartum period, possibly due to a small sample size, the literature suggests that using different cutoff values during pregnancy and the postpartum period may be the best alternative, although there is no consensus on a set cutoff value. While one study suggests a CO cutoff of 2-3 ppm, other studies recommend a 4-ppm breath CO cutoff to identify pregnant smokers [24]. Studies reporting these cutoff values were based on self-report of smoking, which is not always a reliable measure [33-35]. The reported variability in cutoff values is endogenous to exhaled CO as a biomarker since it depends on the given environment in which the measurement is taken [36], an individual’s physiology [11], the breath sampling procedure, and the breath CO measurement instrument [37]. Further validation on optimal breath CO cutoff levels is needed as well as systems that are flexible to adapt to cutoff changes through the perinatal period.

### Limitations and Future Work

This study does not report on the success of financial incentives to prevent smoking relapse but rather on the validity of our measuring instrument and the participant’s experience in using the MoMba Live Long system in person and remotely after a period of 3 months. Future publications will report on a randomized controlled trial on the prevention of smoking relapse using remotely delivered financial incentives. While participants reported that the system was easy to use, there are many unknowns regarding why participants missed remote breath tests. Additional research should investigate reasons for noncompletion, such as difficulty accessing their phone or the required sensor, recent smoking, the timing in which notifications were received, and the social context in which participants are asked to take a test. Prior work has reported some of these factors as negatively impacting adherence to remote dietary interventions [38].

Another limitation of the CO meter is that many other environmental factors can affect the readings of a remote CO monitor, such as air pollution, secondhand smoke, or the use of tetrahydrocannabinol (THC) or cannabis. Expired CO air from THC users has shown to double CO concentration levels [39]. Our sensor is susceptible to increased CO readings based on these external factors. Studies looking into enabling remote smoking detection should consider other substance use screening, as well as assessing a participant’s environment to be aware of possible confounding factors.

### Conclusions

The MoMba Live Long system is one of the first portable breath CO monitoring systems delivering remote CM smoking cessation interventions for pregnant women. The results from this study suggest that CO estimates derived from a smartphone-based breath CO meter are reliable and valid, but further testing in remote and diverse settings is needed to fully understand what environmental and usability barriers may impact the process of taking remote breath tests. Overall, the MoMba Live Long system is a feasible and acceptable approach to help practitioners and researchers increase access and delivery of CM smoking cessation interventions remotely to diverse populations.

## Abbreviations

AUC: area under the curve
CM: contingency management
CO: carbon monoxide
HELLP: hemolysis, elevated liver enzymes, low platelet count
NIH: National Institutes of Health
ppm: parts per million
THC: tetrahydrocannabinol
